# Shorter sleep among adolescents is associated with lower fruit and vegetable consumption the following day

**DOI:** 10.1186/s12966-023-01420-6

**Published:** 2023-02-07

**Authors:** Eleanor M. Winpenny, Harriet Rowthorn, Stefanie Hollidge, Kate Westgate, Ian M. Goodyer, Soren Brage, Esther M. F. van Sluijs

**Affiliations:** 1 MRC Epidemiology Unit, University of Cambridge, Cambridge, UK; 2 Developmental Psychiatry, Department of Psychiatry, University of Cambridge, Cambridge, UK

**Keywords:** Adolescent, Diet quality, Energy density, Longitudinal, Sleep duration, Sleep timing, Fruit and vegetable intake

## Abstract

**Background:**

Insufficient sleep has been associated with weight gain and metabolic dysregulation, with one suggested mechanism being through reduction in diet quality. Experimental evidence supports a causal effect of sleep timings on diet but this may not be applicable to a free-living adolescent population. In this analysis we use daily measures of sleep timings and diet quality, to examine the effect of sleep duration and timing on diet quality the following day among free-living adolescents.

**Methods:**

The ROOTS study is a prospective cohort recruited from secondary schools in Cambridgeshire and Suffolk (UK). Participants (*n* = 815) at mean age 15.0y (SD 0.3y) completed a diet diary and wore a combined heart rate and accelerometer device over 4 consecutive days. Sleep duration and timing (midpoint) were derived from acceleration and heart rate traces, while daily energy density and fruit and vegetable intake were calculated from dietary data. Analyses were performed at day-level (1815 person-days). Multilevel random effects models were used to test associations between sleep each night and subsequent day diet, with daily sleep and diet measures nested within individuals and schools, and adjusted for day-level and individual-level confounding variables.

**Results:**

Adolescents slept a mean of 7.88 hrs (SD 1.10) per night, reporting a mean energy density of 2.12 kcal/g (SD 0.48) and median energy-adjusted daily fruit and vegetable intake of 137.3 g (IQR 130.4). One hour shorter sleep duration was associated with lower intake of fruit and vegetables (-6.42 g, 95%CI -1.84, -10.99) the following day. An association with higher dietary energy density (0.016 kcal/g, 95%CI 0.034, -0.002) the following day was observed but did not reach statistical significance. Sleep timing was not associated with either fruit and vegetable intake (-2.52 g/d, 95%CI -7.66, 2.62) or dietary energy density (-0.001 kcal/g, 95%CI -0.022, 0.020).

**Conclusions:**

Our observational findings from a free-living adolescent population support the experimental evidence for a causal role of sleep on diet, with shorter sleep duration at night leading to a small decrease in diet quality the following day. These findings support experimental evidence to suggest inclusion of sleep duration as one component of interventions designed to improve diet quality and weight status in adolescents.

**Supplementary Information:**

The online version contains supplementary material available at 10.1186/s12966-023-01420-6.

## Introduction

There is substantial evidence that insufficient and poor quality sleep are associated with risk factors for cardiometabolic disease, including weight gain and metabolic dysregulation, in both children and adults [1–5]. Among adolescents in particular, sleep duration is often lower than recommended [6]. A developmental shift in circadian preference (‘chronotype’) to a later sleep and wake time is thought to take place during adolescence [7], which can lead to ‘social jetlag’ [8], whereby societal demands (e.g. early school start times [9]) lead to a mismatch between an individual’s circadian clock and their sleep/wake times. This has been suggested to be exacerbated by high caffeine intake and prolonged use of electronic media in this age group [10].

A recent meta-analysis of prospective studies reported that among adolescents short sleep was associated with a greater risk of developing overweight or obesity (RR:1.30; 95% CI 1.11 to 1.53) [3]. One suggested mechanism for these associations is through poor diet quality and increased energy intake [11], as well as reduced energy expenditure [12, 13]. A number of epidemiological studies in children and adolescents have reported that shorter sleep duration or later bedtimes are cross-sectionally associated with greater consumption of high-energy foods [14, 15], added sugar, and sugar-sweetened beverages [16, 17] as well as lower consumption of nutrient-rich foods like fruits and vegetables [13, 15, 18], leading to poorer overall diet quality [19, 20]. There have also been indications from cross-sectional analyses that a later chronotype (late to sleep and late to wake) may be linked to poorer diet quality and greater adiposity [19, 21, 22]. However, a recent synthesis of the epidemiological evidence has found the evidence to be variable and inconclusive [23]. Given the cross-sectional nature of most of these studies, causal inference is difficult. Rather, associations may be confounded, for example driven by a shared genetic aetiology [24] or behavioural clustering [25].

Meanwhile, experimental sleep deprivation trials undertaken in adults support a causal association between sleep duration and increases in dietary energy intake and weight gain [26, 27]. A meta-analysis of eleven intervention studies found that partial sleep deprivation, typically restricting sleep to between 3 and 6 hrs per night over a period of 1–5 nights, increased energy intake by an average of 385 kcal per day over the same period compared to the control condition [28]. Suggested mechanisms include changes in hunger and appetite-regulating hormones, with changes in leptin and ghrelin levels seen among participants with habitual short sleep duration [29] or under sleep restriction [30, 31]. These hormones increase hunger and appetite, particularly for calorie-dense foods [30]. Trials of sleep deprivation have generally not reported on diet quality, providing dietary analysis at macronutrient level only [28]. Meta-analysis of macronutrient intakes has shown an increase in fat intake and decrease in protein intake under sleep deprivation conditions [28]. There have been fewer experimental studies conducted in adolescents. One experimental study of adolescents aged 14-16y found that sleep restriction (to 6.5 hrs/night) over 5 nights resulted in a higher dietary glycaemic index and more than doubled the intake of sweet/dessert foods compared to a healthy sleep duration (10 hrs/night) [32]. However, these experimental studies are undertaken in laboratory conditions and typically introduce more severe sleep restriction than would be seen in usual life, with food choice restricted to the options provided by the study team [33]. Unless such findings can be replicated in free-living individuals they may not be applicable to population health.

Our aim in this study was to explore whether the hypothesised causal associations between sleep duration/timing and diet quality could be observed in a non-experimental, community-living population. One approach which allows stronger causal inference while maintaining the real-life setting of a free-living population is to study daily variations in diet and sleep patterns within individuals to assess longitudinal influences over consecutive days. This allows identification of longitudinal associations that are not driven by behavioural clustering or individual-level confounding. In this study we examine the possible causal effects of sleep duration and timing on diet quality in a free-living adolescent population. We focus on diet quality, rather than total energy intake, in acknowledgement of the limitations of self-report methods in estimating total energy intake [34], and use two measures of diet quality appropriate for daily intakes: (1) energy density of food, and (2) fruit and vegetable intake. We make use of detailed day-level data including device-measured sleep timings and daily diet diaries to address the research question: What are the causal effects of sleep duration and timing on dietary energy density and fruit and vegetable intakes the following day, among adolescents?

## Subjects and Methods

### Study population and design

The ROOTS study is a prospective cohort study examining the development of psychopathology in adolescence [35]. Participants (*n* = 1238) were recruited via 18 secondary schools across Cambridgeshire and Suffolk, UK from 2005 to 2006. At baseline, when participants were mean age 14·5 years (SD 3·5 months), demographic and psychosocial measures were self-reported. Six months later, a sub-study (*n* = 926) was conducted focussing on diet, physical activity and body composition, in which participants completed a diet diary and simultaneously wore an activity monitor uninterrupted for four consecutive days. The study was approved by the Cambridge University Research Ethics Committee (reference number 03/302). Written informed consent was obtained from all participants and their parents/legal guardians.

### Sleep timings and duration

Participants wore a combined heart rate and movement sensor (Actiheart, CamNtech Ltd., Papworth, UK) to measure habitual activity for the same four consecutive days as their dietary assessment. Prior to monitor wear, heart rate was calibrated using a sub-maximal step-test and monitors were set to record data every 30 seconds. The monitor was fitted to the participant’s chest with two ECG electrodes and s/he was requested to wear the monitor for 4 days and nights without interruption. The full monitoring protocol can be found elsewhere [36].

A single researcher, blind to all other data, reviewed the daily plots of accelerometer and heart rate data to mark sleep onset and sleep end times, as described previously [37]. In accordance with previous research, sleep onset was defined as the beginning of sustained low movement accompanied by a steady decline in heart rate and sleep end was defined as re-initiation of movement after a long period of little-to-no movement, together with an abrupt elevation in heart rate [38]. Daily sleep duration (in hours) was calculated as the difference between sleep termination and onset. The midpoint of sleep (defined as sleep onset time plus half sleep duration) was used as a continuous measure of sleep timing, with a later midpoint representing a later sleep and wake time [39]. Where sleep onset and sleep end times could not be ascertained from the activity plots, these cases were excluded. Prior to assessing sleep timings on this dataset, the researcher completed a training dataset (*n* = 20) discussing any discrepancies with a physical activity measurement specialist.

### Dietary energy density and fruit and vegetable intake

Participants were provided with a 4-day diet diary to complete, which included two weekdays and two weekend days. Participants were asked to report estimated portion sizes in terms of small, medium or large, household measures or as individual items. Training was provided to participants, which involved practice diary completion and feedback from the research team. On return of completed diary and physical activity monitor, participants received a £30 voucher. Diets were coded at the Medical Research Council (MRC) Human Nutrition Research Unit (Cambridge, UK) using the Diet-In-Nutrients-Out (DINO) system [40]. Portion weights were approximated using published values for children [41–43].

The dietary outcome measures in the present study were daily energy density of food (ED), and daily energy-adjusted fruit and vegetable intake. Both measures are established as proxies for diet quality; low ED is associated with higher quality diets that contain a greater proportion of fruits and vegetables [44, 45], while high ED has been associated with weight gain and adiposity among children and adults [46, 47]. Daily ED was calculated as total kcals/total grams of food consumed (excluding beverages) [48, 49]. Fruit and vegetable intake was adjusted to an energy intake of 1800 kcals/d using the residual method [50, 51].

We considered any day when any food intake was reported to be a valid day, without imposing energy intake criteria. Although some studies exclude those with very high or low reported energy intakes from dietary analyses, energy-intake cut-offs are applied to average dietary intake assessed over several days, rather than daily intakes which are naturally more variable [52].

### Covariates

Data on demographic covariates were collected at baseline. Participants self-reported their sex. Neighbourhood-level socioeconomic status was assessed using the ACORN index to categorize UK postcodes into five categories [53]. These categories were further collapsed to give three categories: high (categories 1/2), medium (category 3) and low SES (categories 4/5). Height (in metres) and weight (in kilograms) were measured during the school visit for the diet and activity sub-study, by trained research assistants following standard protocols. BMI z-score (zBMI) was calculated based on reference curves for the UK [54].

Depressed mood was assessed at baseline using the Moods and Feelings Questionnaire (MFQ) [55], a 33-item self-report measure of depressive symptoms, including factors such as low mood, loss of appetite, anhedonia, irritability and restlessness. Participants were asked to report their mood over the previous 2 weeks against each item on a three-point scale (mostly/sometimes/never), giving an overall score ranging from 0 to 66. The MFQ has moderate to high criterion validity as a screen for adolescents with unipolar depression, with an optimal cut-point of ≥20 on the MFQ suggested for discriminating participants with any mood disorder from those with no mood disorder and an optimal cut-point of ≥29 on the MFQ suggested for identifying participants having current major depressive episodes [56].

Physical activity (PA) was measured using the combined heart rate and movement sensor (Actiheart, CamNtech Ltd., Papworth, UK) as described above for sleep assessment. Heart rate data was cleaned, individually calibrated and Physical Activity Energy Expenditure (PAEE) was estimated by branched equation modelling as described previously [36, 57]. Valid monitor wear to determine daily PAEE was defined as ≥12 h of data, including at least 2 hours of data from each quadrant of a 24 h day (morning (03.00–09.00 hours), noon (09.00–15.00 hours), afternoon (15.00–21.00 hours) and night (21.00–03.00 hours)). This inclusion criteria minimised the potential bias that could result from under or over-representation of data if the monitor was only worn at specific times of the day. PAEE (kJ/kg/day) on the day prior to the night of sleep was included as a covariate that could affect both sleep and dietary choices the next day. Due to the design of the study, PA data was only available on day one from the time when the sensor was activated during the school visit. We therefore imputed missing hours of PA data from quadrants one and two of day one, derived as the mean of values from the same hour of data collected on the remaining study days, in order to be able to include the first day in PAEE-adjusted analyses.

### Statistical analysis

Statistical analyses were conducted in Stata/SE 16.1 (StataCorp, College Station, TX, USA). Student’s t tests and χ2 tests were used to assess differences in sociodemographic and anthropometric variables between those included and excluded from the current analysis, and between boys and girls.

**Fig. 1:**
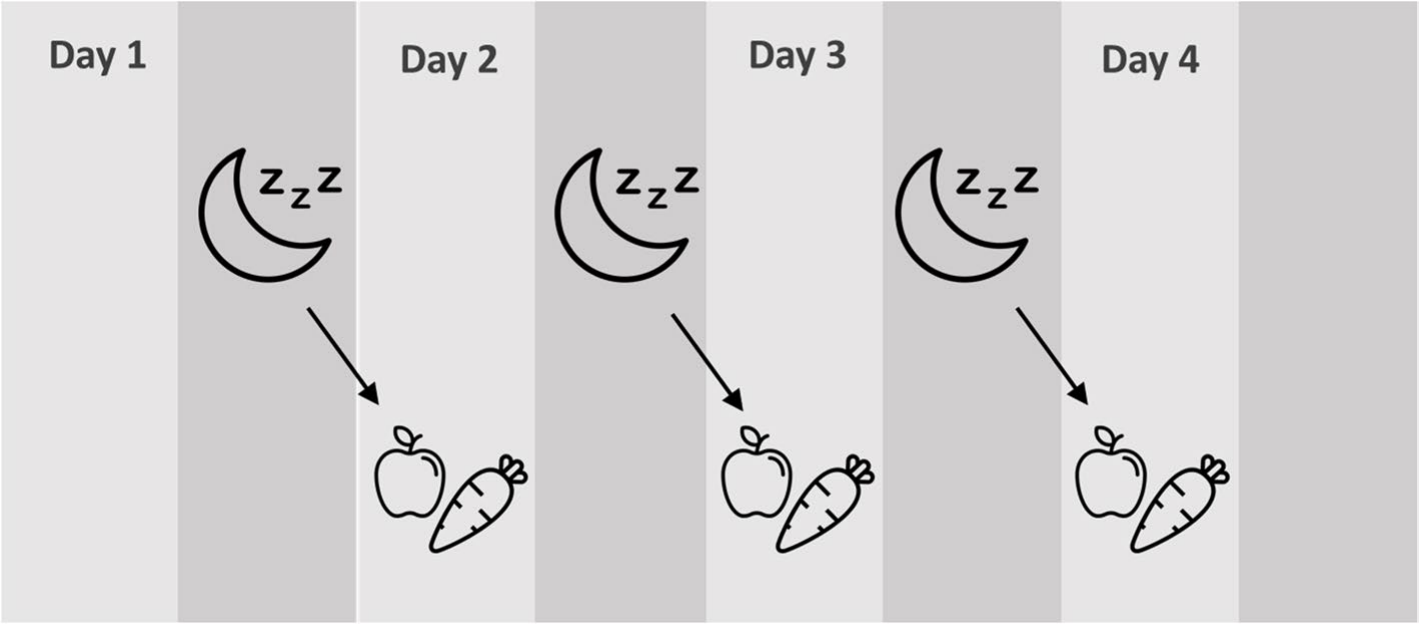
A schematic showing day-level associations between sleep at night and subsequent day diet. Physical activity energy expenditure on the previous day, dietary intake variables the previous day, and day of the week were included as day-level covariates, and sex, age, ACORN-SES and depressed mood were included as person-level covariates

Association analyses were conducted at person-day level. Models were fitted to the data to assess the association of two properties of sleep (sleep duration and sleep midpoint) with two measures of diet quality on the subsequent day (energy density of food consumed and fruit and vegetable intake). Multilevel, random effects models were used to test associations between daily sleep and diet measures (level 1) nested within individuals (level 2), and within schools (level 3). A schematic of this day-level analysis is shown in Fig. 1 and a more detailed Directed Acyclic Graph is provided as Supplementary Fig. 1. Models were built hierarchically, as recommended by Twisk [58], with the same modelling strategy applied for each outcome variable. First, an empty model containing only a fixed intercept was fitted to the data and this was compared to a 3-level random effects model that allowed intercepts to vary across participants, nested within schools. This empty 3-level model was used to investigate the partition of variance between the three levels. The fixed effects of the primary predictors (sleep duration and timing) were then added to the model and the model fit was assessed. We tested a random slope model to determine if the nature of the relationship between sleep duration/timing and next-day diet differed across individuals, however this did not improve model fit (assessed using a likelihood ratio test), so we reverted to a random intercept model. We tested inclusion of a limited number of level 2 (individual level) covariates in our models. Sex, age, ACORN-SES zBMI and MFQ were tested as potential confounding variables based on past research showing that these factors are associated with both sleep duration and diet quality [59–61]. Of these, sex, age, Acorn-SES, and MFQ improved model fit and were included in the models. At level 1 (day level) the day of measurement and physical activity energy expenditure (PAEE) on the previous day were included as confounding variables, since these might influence both sleep duration/timing and diet quality. We considered inclusion of alcohol intake (as reported in the diet diary) as a level 1 covariate, however reported alcohol intake was very low in this cohort (age 15y), so was not included. We also tested inclusion of the dietary outcome variables from the previous day (dietary energy density or fruit and vegetable intake) and other relevant dietary variables from the previous day (intake of caffeinated beverages, and sugar-sweetened beverages). These made small contributions to the models so were included as confounding variables. We tested whether sleep duration models should be adjusted for sleep mid-point and sleep mid-point models adjusted for sleep duration, but such mutual adjustment did not improve model fit, suggesting that these exposures function independently and do not confound one another.

## Results

Of the 1238 individuals recruited into the ROOTS cohort at baseline, 926 participants (75%) completed the dietary sub-study. Of these, 872 (70% of baseline) had valid sleep data, 832 (67%) had valid PA data, and 815 (66%) had baseline mood data. These 815 individuals provided 1815 days of data on sleep and diet, with a mean of 2.23 (SD 0.9) days of data per individual.

The population included in the dietary sub-study included a slightly higher proportion of girls than the excluded population (56.8% vs 49.9% female, *p* < 0.05) but showed no significant differences in age (14.5y (SD 0.3) vs 14.5y (SD 0.3)) or Acorn SES (62.3% high, 23.6% medium, 14.1% low SES vs 60.0% high, 25.3% medium, 15.1% low SES, *p* = 0.68) from the excluded population. Descriptive data on the included study population are shown in Table 1. These data reveal that mean sleep duration, as assessed by the combined heart rate and movement sensor, was low compared to national recommendations [62] and lower among boys than girls. Diet quality was also poorer on average among boys than girls, reflected as a higher energy density of food, and lower fruit and vegetable intake.

**Table 1:** Demographic characteristics of the included sample

		All participants (*n* = 815)	Girls (*n* = 463)	Boys (*n* = 352)
Sex, % female		56.5	–	–
Acorn SES, %	High	62.3	62.9	61.7
	Medium	23.6	24.4	22.4
	Low	14.1	12.7	15.9
Age at dietary sub-study, years, mean (SD)		15.0 (0.3)	15.0 (0.3)	15.0 (0.3)
BMI z-score (SD)		1.04 (0.17)	1.05 (0.17)	1.03 (0.17)
Mean daily sleep duration, hrs, mean (SD)		7.88 (1.10)	8.06 (1.13)	7.66 (1.00)***
Mean timing of sleep midpoint, hrs:mins, mean (SD)		03:49 (1:02)	03:53 (1:03)	03:44 (1:01)*
Reported energy intake, kcals, mean (SD)		1796 (614)	1649 (552)	1988 (638)***
Energy density of food, kcal/g, mean (SD)		2.12 (0.48)	2.09 (0.48)	2.16 (0.47)*
Fruit and vegetables (energy adjusted to 1800 kcals/d), grams, median (IQR)		137.3 (130.4)	147.8 (132.0)	123.6 (127.1)**
PAEE, kJ/kg/day (SD)		76.3 (27.1)	69.1 (24.7)	85.6 (27.3)***

Test of statistically significant differences between male/female sample: *p < 0.05, **p < 0.01, ***p < 0.001

Partition of variance of diet variables into within-person, between-person and school-level variance in a 3-level model revealed high within-person variance, with 78% of the total variance in energy density attributed to within-individual variation, 20% between-person variation and 2% school level variation. Similarly, 61% of the variance in fruit and vegetable intake was within-person, with 35% between-person and 4% at school-level.

Table 2 shows that longer sleep duration was associated with both lower energy density of food and higher fruit and vegetable intake, although the 95% confidence interval for the association of sleep duration with energy density overlapped zero following covariate adjustment. Sleep timing, as assessed using the mid-point of sleep, was not associated with either energy density or fruit and vegetable intake.

**Table 2:** Associations between daily sleep duration and sleep midpoint and subsequent day dietary energy density and fruit and vegetable consumption (beta coefficients and 95% confidence intervals)

	Unadjusted (*n* = 832 participants, *n* = 1855 days)		Adjusted^a^ (*n* = 832 participants, *n* = 1855 days)	
	Energy density (kcal/g)	Fruit and vegetable intake (g)	Energy density (kcal/g)	Fruit and vegetable intake (g)
Sleep duration (hrs)	-0.024 (-0.04, -0.005)	7.86 (3.35, 12.37)	-0.016 (-0.034, 0.002)	6.42 (1.84, 10.99)
Sleep midpoint (hrs)	-0.003 (-0.023, 0.016)	-2.90 (-7.84, 2.05)	-0.001 (-0.022, 0.020)	-2.52 (-7.66, 2.62)

^a^Adjusted models were adjusted for sex, SES, age depressed mood, day of measurement, previous day PAEE, dietary outcome the previous day, caffeinated beverage intake and sugar-sweetened beverage intake the previous day

## Discussion

This study estimated the effect of sleep duration and timing on diet quality the following day, based on data collected from a free-living adolescent population. Our findings from this analysis suggest that a shorter sleep duration at night leads to poorer diet quality the following day, exemplified in particular by a reduced intake of fruit and vegetables. The effects seen were independent of person-level behavioural clustering and confounding variables, although effect sizes were small. By contrast, we found that earlier or later sleep timing did not show associations with diet quality the following day.

These findings build on existing experimental and epidemiological evidence [23, 28], by using a new approach to provide further support for a causal association between sleep and diet that has relevance to everyday life [63]. Previous epidemiological research has been largely limited by weak measures of sleep and dietary intake and cross-sectional study designs, with inconsistent findings on the association between sleep and diet [23]. While experimental studies of imposed sleep deprivation have shown that the association is likely to be causal [28], it is unclear whether these effects would apply in relation to the patterning and level of variation in sleep duration which occurs in a free-living adolescent population. To our knowledge this is the first study to assess sequential day-to-day associations between sleep duration and timing and next day diet quality in a free-living population, making use of multilevel models to take advantage of the natural variance from primarily within-person effects. Triangulation of our findings with previous epidemiological and experimental evidence, provides confidence to suggest a causal effect of sleep duration on diet quality which extends to free-living populations [63].

The aim in this study was to provide real-world support for the causal associations between sleep and diet observed in experimental studies, rather than estimate the likely influence of this association on public health. As we discuss further below, interpreting public health implications based on changes observed over a single day is problematic. However, we note that the size of the associations seen in this study were small. One hour shorter sleep duration was associated with a subsequent 0.02 kcal/g higher energy density, which equates to only 4% of the standard deviation of energy density in this population. Similarly the observed 6 g lower fruit and vegetable intake is small relative to a median daily fruit and vegetable intake of 137.3 g (IQR 130.4) in our sample, and a recommended daily intake of 400 g [64]. Nevertheless, some have argued that small changes to diet quality and dietary energy density can have a meaningful effect on weight gain over time [65].

### Strengths and limitations of the study

The strengths of this study lie in the detailed analysis of real-life day-level variation in sleep timings and diet quality to assess daily associations between sleep and diet. An important strength was the high-quality data available on sleep timings and dietary intake, collected at a daily level. Multi-day diet diaries are one of the most robust methods of collecting dietary data from adolescents [66], and are often used to establish dietary patterns or diet quality scores as a measure of habitual diet [67]. To assess diet quality at a daily level, we opted not to use diet quality scores since these measure a wide range of items which are not likely to be consumed on a single day. Instead, we made use of two measures of diet quality which can be assessed on a daily basis, namely energy density and fruit and vegetable intake. These measures are associated with broader diet quality [44], as well as adiposity [46, 47] and physical and mental illness [68–70].

We used a combined heart rate and movement sensor to assess sleep exposures. The gold-standard for sleep assessment is polysomnography which includes heart rate. Measurements of body movement have been validated over many studies against the gold-standard of sleep measurement polysomnography [71–73], and have gained clinical acceptance as a valid method for measurement of sleep parameters in free-living patients [74]. Combining accelerometry with heart rate measurement provides an additional data source for sleep timing estimation. Measurement with a sensor is less prone to inaccuracies and bias compared with to self-report [75]. Moreover, the use of a sensor allows measurement of sleep timings over several days, and is less burdensome than completion of a sleep diary. While there is some subjectivity in the classification of sleep timings from accelerometer and heart rate traces, the completion of these assessments by a single researcher, blind to all other data means that this step may add some error but not generate bias.

It is important to acknowledge that we have only examined short-term associations, from one night to the subsequent day. This study therefore does not provide evidence of how short sleep duration may contribute to changes in diet over a longer time period. It is possible that compensation of dietary intakes may also occur over a longer time period, with those individuals that show a higher dietary energy density on a given day, compensating through reduced dietary energy density on subsequent days. Alternatively, it may be that the effects of sleep deprivation on diet become more severe as sleep deprivation becomes chronic. Others have argued for a more complex cyclical relationship between sleep and diet [76]. As stated, our aim in this piece of work was not to estimate the overall impact that short sleep duration might have over an extended period of time, but to assess if the evidence from experimental studies would be observable in the more immediate short-term effect of sleep on diet, in a free-living adolescent population. One limitation of this dataset is that we were not able to adjust for day-level measures of mood or stress which may influence health-related behaviours such as sleep and diet. However, we believe it is unlikely that fluctuations in mood would be on the same daily timescale as the night-day sleep-diet associations studied here. Adjustment for baseline MFQ and the individual-level random intercept in the models accounts for longer-term differences in mood between individuals.

The ROOTS study was not designed to be representative of a particular population, but rather to sample a broad range of adolescents from the counties of Cambridgeshire and Suffolk. Compared with national UK data, the ROOTS cohort includes a higher proportion of participants from higher SES categories [35]. However, given the suggested biological mechanisms for the influence of sleep on diet, including the influence of appetite-regulating hormones [31], we suggest that these findings are likely to be consistent across different populations.

## Conclusion

Our findings in this study support the experimental evidence of a causal effect of sleep on diet, with a shorter sleep duration associated with a decreased fruit and vegetable intake the following day. Importantly identification of these associations in an observational cohort study suggests that such mechanisms are relevant at the level of day-to-day sleep variation seen in the general adolescent population, although the effect sizes seen are small. Our findings support the need to consider sleep as a determinant of a healthy diet, and inclusion of sleep duration as one component of interventions designed to improve diet quality.

## Supplementary Information

**Additional file 1: Fig. S1.** A directed acyclic graph depicting day-level (blue) and person-level (green) confounders of the sleep-diet relationship.

## Data Availability

Data are available in a public, open access repository. EW had full access to all the data in the study and takes responsibility for the integrity of the data and the accuracy of the data analysis. The data are deposited in the University of Cambridge Data Repository, doi.org/10.17863/CAM.82688. Data are available upon reasonable request to researchers via openNSPN@medschl.cam.ac.uk.

## Abbreviations

CI: Confidence interval
DINO: Diet-in-nutrients-out
ECG: Electrocardiogram
ED: Energy density
g: Gram
hrs: Hours
IQR: Interquartile range
kcal: Kilocalories
kg: Kilogram
kJ: Kilojoules
MFQ: Mood and feelings questionnaire
MRC: Medical research council
PA: Physical activity
PAEE: Physical activity energy expenditure
SD: Standard deviation
SES: Socioeconomic status
UK: United Kingdom
y: Years
zBMI: Body mass index z-score
