# Supplementary figures and images for: Shorter sleep among adolescents is associated with lower fruit and vegetable consumption the following day
Source: Int J Behav Nutr Phys Act. 2023 Feb 7;20:12. doi: 10.1186/s12966-023-01420-6 (PMC9906927; doi:10.1186/s12966-023-01420-6)

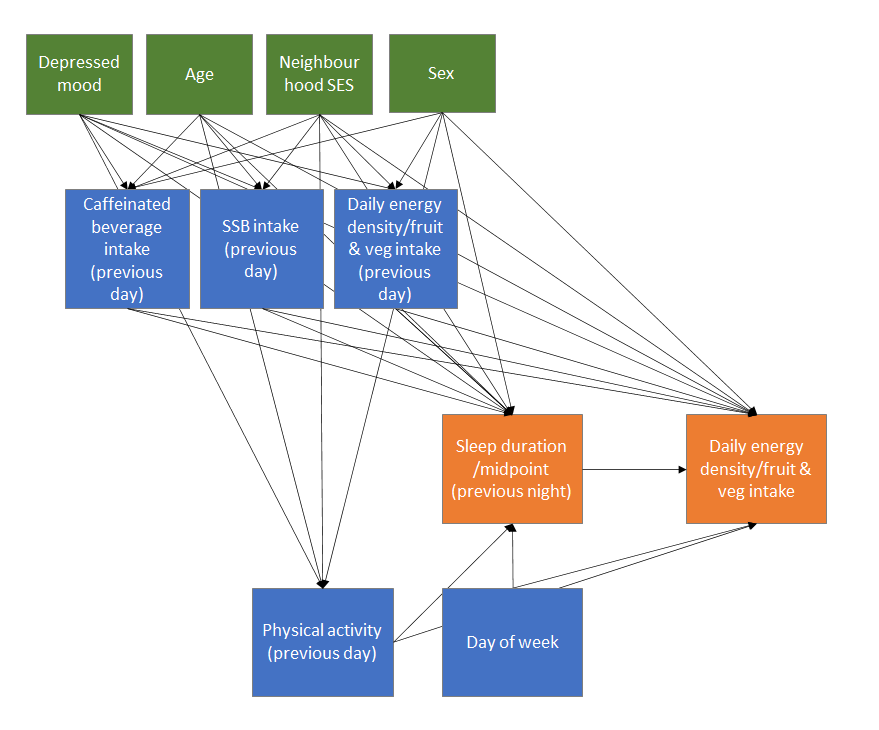

Supplement: Supplementary file 1 — Additional file 1: Fig. S1. A directed acyclic graph depicting day-level (blue) and person-level (green) confounders of the sleep-diet relationship. [file 12966_2023_1420_MOESM1_ESM.tif]
